# The Barcelona Brain Health Initiative: A Cohort Study to Define and Promote Determinants of Brain Health

**DOI:** 10.3389/fnagi.2018.00321

**Published:** 2018-10-11

**Authors:** Gabriele Cattaneo, David Bartrés-Faz, Timothy P. Morris, Javier Solana Sánchez, Dídac Macià, Clara Tarrero, Josep M. Tormos, Alvaro Pascual-Leone

**Affiliations:** ^1^Institut Guttmann, Institut Universitari de Neurorehabilitació adscrit a la UAB, Badalona, Spain; ^2^Institut d’Investigacions Biomèdiques August Pi i Sunyer, Barcelona, Spain; ^3^Departament de Medicina, Facultat de Medicina i Ciències de la Salut, Universitat de Barcelona, Barcelona, Spain; ^4^Universitat Autònoma de Barcelona, Barcelona, Spain; ^5^Fundació Institut d’Investigació en Ciències de la Salut Germans Trias i Pujol, Badalona, Spain; ^6^Berenson-Allen Center for Noninvasive Brain Stimulation, Division of Cognitive Neurology, Department of Neurology, Beth Israel Deaconess Medical Center, Harvard Medical School, Boston, MA, United States

**Keywords:** brain health, lifestyles, cognitive reserve, multi-dimensional, intervention, cognitive enhancement, cohort study

## Abstract

The Barcelona Brain Health Initiative (BBHI) is an ongoing prospective longitudinal study focused on identifying determinants of brain health. The main objectives are: (i) to characterize lifestyle, cognitive, behavioral and environmental markers related to a given individual’s cognitive and mental functions in middle to old age, (ii) to assess the biological determinants predictive of maintenance of brain health, and (iii) to evaluate the impact of a controlled multi-dimensional lifestyle intervention on improving and maintaining brain health. The BBHI cohort consists of >4500 healthy participants aged 40–65 years followed through online questionnaires (Phase I) assessing participants’ self-perceived health and lifestyle factors in seven different domains: overall health, physical exercise, cognitive activity, sleep, nutrition, social interactions, and life purpose. In Phase II a sub-group of 1,000 individuals is undergoing detailed in-person evaluations repeated at two-yearly intervals. These evaluations will provide deep phenotyping of brain function, including medical, neurological and psychiatric examinations, assessment of physical fitness, neuropsychological assessments, structural and functional brain magnetic resonance imaging, electroencephalography and perturbation-based non-invasive brain stimulation evaluations of brain activity, as well as collection of biological samples. Finally, in Phase III a further sub-group of 500 participants will undergo a similar in-person assessment before and after a multi-dimensional intervention to optimize lifestyle habits and evaluate its effects on cognitive and brain structure and function. The intervention group will receive remote supervision through an ICT-based solution, with the support of an expert in health and lifestyle coaching strategies aimed at promoting adherence. On the other hand, the control group will not have this coaching support, and will only receive education and recommendations about healthy habits. Results of this three-part initiative shall critically contribute to a better understanding of the determinants to promote and maintain brain health over the lifespan.

## Introduction

Human life expectancy has significantly increased in recent decades thanks to the advances in public health and medicine. In the developed world the number of older adults (>65 years) has already surpassed the number of children (<15 years), and by 2050 the proportion of elderly people is projected to nearly double that of the young (United Nations, 2017). However, the extension of lifespan does not correlate well with the extension of a healthy lifespan (or health-span; Passarino et al., 2016).

Advancing age is the major risk factor for the development of neurological and psychiatric brain disorders, and aging is associated with increased prevalence of conditions such as epilepsy, stroke, and major neuropsychiatric or neurodegenerative diseases (Barnett et al., 2012). Epidemiologic data collected in recent decades indicate the strong need to focus research efforts on the prevention of these disorders, given that their prevalence is greater than that of cardiovascular diseases and cancer, representing 13% of the global burden of diseases (Collins et al., 2011). In Europe each year almost 40% of the population suffers from at least one disorder pertaining to the neuropsychiatric spectrum (Wittchen et al., 2011), and according to World Health Organization (WHO) estimations, by 2030, half of the world-wide economic impact of disability will be due to brain related disability (as measured by disability-adjusted life year, DALY; Mathers and Loncar, 2006).

Brain Health is defined as the development and preservation of optimal brain integrity and neural network functioning for a given age (Gorelick et al., 2017). Maintaining brain health across the lifespan is likely linked to preservation of efficient mechanisms of plasticity, i.e., the nervous systems’ ability to make rapid adaptations to changeable internal and external environmental demands (Pascual-Leone, 2006). The mechanisms of plasticity evolve over the lifespan but remain responsive to changing environmental factors even at advanced ages. Alterations in the mechanisms of plasticity are related to the development of disease or disability (Pascual-Leone et al., 2011). However, impaired efficacy, and a resulting loss in brain health, does not appear to be an obligatory consequence of aging (Pascual-Leone et al., 2011).

Longitudinal studies tracking brain health metrics, such as general cognitive (Yaffe et al., 2009) and memory performance (Josefsson et al., 2012), suggest three main trajectories of cognitive aging: (1) individuals who exhibit significant cognitive decline over time; (2) individuals who show cognitive decline yet remain within an age-appropriate range; and finally (3) individuals who maintain cognitive performance and experience little cognitive decline even at advanced age. Similarly, some individuals show steep decrements of the efficacy of the mechanisms of plasticity over time, whilst others do not (Pascual-Leone et al., 2011; Freitas et al., 2013). Theoretical models such as ‘cognitive or brain reserve’ (Stern, 2009) or ‘brain maintenance’ (Nyberg et al., 2012) have been put forward to better understand the individual characteristics and underlying brain mechanisms associated with preservation of cognition and brain function in the face of advancing age or even the initial stages of pathology. Beyond theoretical models, neuroimaging studies have stressed that the preservation of cognitive and mental health is associated with either the maintenance of brain structure, activity or connectivity patterns with advancing age (Nyberg et al., 2012), and/or with the expression of compensatory responses in the face of pathological changes (Stern, 2012; Elman et al., 2014; Reuter-Lorenz and Park, 2014).

In recent years a major focus has been placed on the discovery and validation of new ‘biomarkers’ for major diseases (Olsson et al., 2016 for a recent review and meta-analysis) that allow the identification of ‘at risk’ individuals, or even subjects at ‘preclinical stages’ of diseases, years before the possible manifestation of clinical symptoms (e.g., Dubois et al., 2007). It is now well-accepted that structural and functional brain changes associated with the development of neurodegenerative diseases can begin some 10–20 years prior to the onset of symptoms (Beason-Held et al., 2013). During this “pre-clinical period,” individual differences in brain resilience may delay the appearance of symptoms or act to reduce or eliminate the clinical and behavioral impact of pathologies (Nyberg et al., 2012). For this reason, there is a strong need to focus research on such factors that could prevent illness and promote brain resilience in the presence of pathology. For example, in the case of dementia, delaying the onset of symptoms for just 1 year could prevent disability in over 11.8 million cases in the next 30 years representing a cost savings of $219 billion (Brookmeyer et al., 2007; Zissimopoulos et al., 2015).

### Modifiable Lifestyle Factors and Their Interactions With Biological Markers

**FIGURE 1:**
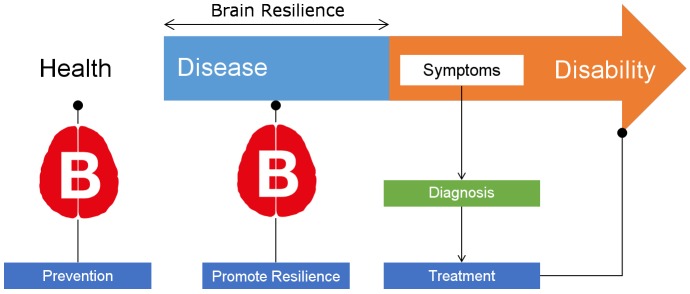
Schematic representation of disease progression and the time frame in which modifiable lifestyles could prevent disease onset or promote brain resilience (see Arenaza-Urquijo and Vemuri, 2018 about the differences between these two terms). The B brain images illustrate where the objectives and hypotheses of the BBHI aim to have the most impact.

Recently it has been proposed that experience-based changes in brain structure and function may act, in aging, as protective factors that contribute to intra-individual differences in the resilience to brain pathologies (Gelfo et al., 2017). Thus it has been suggested that specific modifiable health and lifestyle factors have the potential to prevent and delay the onset of dementia and improve coping or recovery from injuries or illnesses (see **Figure 1**; Di Marco et al., 2014; Frankish and Horton, 2017; Livingston et al., 2017). It has been estimated that several modifiable risk factors, such as physical activity, socialization, weight and blood pressure control, psychological well-being and cognitive activity across the lifespan could prevent more than 30% of the diagnosed cases of dementia (Livingston et al., 2017). Similarly, it has been proposed that specific dietary patterns (e.g., Mediterranean diet; Guasch-Ferré et al., 2017, see Loughrey et al., 2017 for a review) good sleep quality (Sexton et al., 2014; Fung et al., 2016), and definition of a clear purpose in life (Ryff et al., 2016; Wilson and Bennett, 2017) have a positive effect on brain health in aging and may reduce the incidence of brain diseases.

Lifestyles have been shown to interact with biomarkers and have the potential to promote mechanisms of brain resilience (Landau et al., 2012; Wirth et al., 2014; Arenaza-Urquijo et al., 2015). For example, exposure to enriched cognitive environments at a given age may promote maintenance of cognitive function and attenuation of Aβ amyloid deposition among apolipoprotein (APOE) ε4 allele carriers (Vemuri et al., 2014). Similarly, physical activity and adherence to the Mediterranean diet has been shown to attenuate the burden of disease in Alzheimer’s disease (AD) (Matthews et al., 2014), as well as sustain brain structure and cognitive function in older adults (Kramer and Erickson, 2007).

Beyond an association between lifestyle factors and brain health, the potentiation of certain brain health metrics has been evidenced via dedicated interventions. For example, dedicated physical exercise programs have been shown to preserve neural networks associated with cognitive decline (Voss et al., 2016), and dietary interventions (high antioxidant rich foods associated with the Mediterranean diet as well as walnuts) have been shown to improve cognitive function (Valls-Pedret et al., 2017). Consequently, the potential to modify such lifestyle habits associated with brain health exists and may lead to the promotion and modification of brain health across the lifespan (Ngandu et al., 2015; Rosenberg et al., 2018).

However, there remains a significant knowledge gap precluding the personalized prescription of specific lifestyle modifications to promote an individual’s brain resilience and reduce the incidence of major neuropsychiatric and neurological diseases. The Barcelona Brain Health Initiative (BBHI) seeks to fill these knowledge gaps.

### The Barcelona Brain Health Initiative: Rationale

The BBHI is a longitudinal prospective cohort study with the primary aim to examine, characterize and promote the lifestyle factors, biological determinants and their interactions that permit certain individuals to avoid the development or clinical manifestation of major neuropsychiatric and neurodegenerative disorders. The main objectives of the BBHI are (i) to characterize lifestyle, cognitive, behavioral and environmental markers associated with good cognitive and mental functions in middle to old age, (ii) to assess the biological determinants predictive of the maintenance of brain health and prevention of neuropsychiatric illnesses over aging, and (iii) to evaluate the effect of a controlled multi-dimensional lifestyle intervention on improving brain health.

## Materials and Methods

### Design

The BBHI consists of three phases: In **Phase I** volunteers are engaged through the creation of a personal profile in a dedicated web-based platform^1^, and periodically asked to respond to on-line questionnaires and surveys. Frequent email contact, social media interactions and in-person meetings with selected groups of participants help keep the cohort engaged and offer important educational value on lessons learned regarding factors that promote and sustain brain health. In **Phase II**, a sample of 1,000 participants is undergoing a detailed clinical phenotyping through a multi-day in-person evaluation that includes cognitive, physical and medical assessments, biological sample recollection, structural and functional magnetic resonance imaging (MRI), electroencephalography (EEG) and perturbation biomarkers with transcranial magnetic stimulation (TMS) combined with EEG. A critical component of this phase II is the longitudinal, biannually repeated evaluation to identify biologic correlates of subjective and behavioral changes. Finally, in **Phase III**, a sample of 500 participants will undergo a controlled non-pharmacological clinical trial to evaluate the impact of a multi-dimensional intervention designed to improve metrics of brain health including psychological, medical and biological indicators.

### BBHI Cohort

#### BBHI Launching and Recruiting

A comprehensive communication campaign was designed and implemented to publicize the project and recruit participants. After a kick-off press conference, the BBHI project was covered in both local and national media with more than 10 interviews on the mainstream radio and TV programs. The project’s public and private website, as well as social media (Facebook and Twitter accounts), were developed and continue to increase their reach monthly. Furthermore, over 15 large companies were contacted to raise their interest and encourage their workers to register and participate in the project, through conferences and internal communication channels. Conferences for the general public are regularly held in different forums for information as well as recruitment purposes.

In addition, an Ambassador program involving public opinion leaders in the age range of the study participants helps spread the values promoted by the project (healthy diet, physical fitness, etc.). Several well-known personalities from sport, culture and nutrition have participated as BBHI ambassadors through videos of support for the project and media events. Furthermore, testimonies from volunteers have been also recorded, aiming to create a sense of proximity to the target population of the study.

#### The BBHI Community

Beyond the study cohort, and in an effort to more broadly educate the public on determinants of brain health and establish stable platforms to share and disseminate meaningful results, we established the BBHI community. The BBHI community includes the cohort of participants that have signed up to the study via the online web-based platform or mobile application, but also anyone interested in the topic or linked, directly or indirectly, to the BBHI team. In addition to regular communication via social media, periodic public conferences are organized. Through the BBHI community, we disseminate information on recent findings and scientific breakthroughs on the benefits of healthy lifestyle habits, trying to convert validated scientific evidence into brain health-promoting recommendations. Thus the BBHI invests effort into social responsibility. Furthermore, through larger events – such as sports or culinary celebrations – we seek to promote a sense of belonging and stake-holder engagement.

As an important component of the BBHI community, active involvement of the volunteers in the study has been ensured through the designation of a “reference group” of volunteers, representative of the wider cohort, who are periodically invited to face to face meetings with the BBHI research and leadership team. Feedback from the BBHI community is thus an integral contributor to study protocols and procedures.

#### Characterization of the Cohort

Barcelona Brain Health Initiative study participants are community-dwelling individuals between 40 and 65 years of age, of both sexes, free from any self-reported neurological or psychiatric diagnosis at the time of recruitment. As of the end of June 2018, 4,757 participants have enrolled in the project via the web-based application and completed the first on–line questionnaire. Of these, 189 subjects were excluded because they reported to be less than 40 years old or older than 65 years. Another 362 subjects reported to have been diagnosed with neurologic or psychiatric disease, including Alzheimer’s disease, Parkinson’s disease, multiple sclerosis, amyotrophic lateral sclerosis, cerebral stroke, schizophrenia, or major depression. Accordingly, 4,206 participants (2,792 women) are active participants in the study to date. Mean age ± standard deviation of the participants is 52 ± 7 years with an age distribution that is highly representative of the Catalan population. The cohort does, however, include an over-representation of women (66%), and participants have overall a higher education level than the general population (70% have advanced studies, bachelor degree equivalent or higher). The socio-demographic information of currently enrolled participants is summarized in **Table 1**.

**Table 1:** Demographic characteristics of currently enrolled participants.

	Men	Women	

	***Mean (SD)***	***Mean (SD)***	***p*-value**
Age (years)	52.8 (7.3)	52.0 (7.3)	<0.01^∗^

	**Men**	**Women**	**Total**

	***N (%)***	***N (%)***	***N (%)***

Participants	1414 (33.4)	2792 (66.6)	4206 (100)

**Educational status**			
Without studies	1 (0.1)	0	1 (0.0)
Primary	60 (4.2)	116 (4.2)	176 (4.2)
Secondary	402 (28.4)	628 (22.5)	1030 (24.5)
Superiors	951 (67.3)	2048 (73.3)	2999 (71.3)


*^∗^Univariate ANOVA comparing mean of men and women’s age.*

### Phase 1: Self-Report Online Questionnaires

**FIGURE 2:**
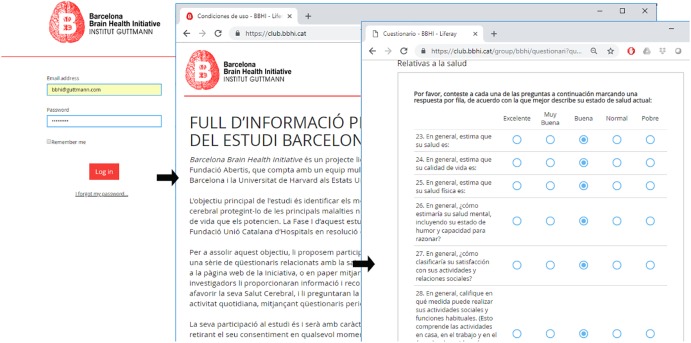
Example of participants’ personal profile page and web-based questionnaires.

All procedures were approved by the ethics and research institutional review board of the Institut Guttmann. Phase I has been designed completely online. Each participant registers in the study through the website of the project^2^, by filling out an initial form. This creates a personal account on the private website of the project^3^, associated to their personal email address. At the first login, after setting a password, participants are prompted with a web-based consent form, explaining the aims of the study, consequences and benefits of participating in this Phase I of the study. Enrollment in the study is completed only after accepting this consent. Subsequently participants can access a personal profile page, where the online questionnaires are available to be completed (see **Figure 2**).

Upon recruitment to the study, and provision of the web-based informed consent, participants are asked to respond periodically to different questionnaires, that describe their self-perceived health and lifestyles in the 7 domains hypothesized to be related to brain health. These 7 “pillars of brain health” are overall physical/general health, nutrition, sleep, physical activity, cognitive activity, socialization and vital plan. Moreover, biographical and socio-demographical information, as well as other factors related to cognitive reserve, are collected. Furthermore, being based in the Catalan city of Barcelona, affords the study the possibility to study the specific contribution of bilingualism. We thus have the opportunity to study the specific contribution of some variables related with bilingualism (proficiency, age of acquisition, use, etc.) to cognitive reserve and brain health (Calabria et al., 2017).

The primary end point of participation in the study is the diagnosis of any brain (psychiatric or neurological) diseases. Therefore, participants are asked to report new diagnosis upon their appearance, and every year we query them for new diagnoses and about the number of times they have visited their GP.

**FIGURE 3:**
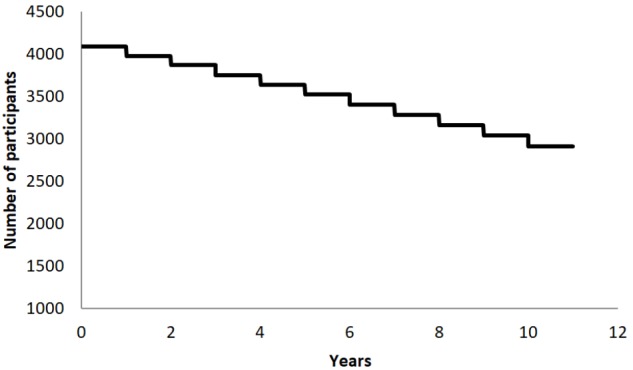
Graphical representation of the expected survival curve (classified by the study’s primary end point; a diagnosis of any psychiatric or neurological disease) of the cohort, calculated based on epidemiological and demographical data of participants.

A literature review of epidemiological data collected in Catalan, Spanish, and European populations regarding major neuropsychiatric diseases and compared with demographical characteristics of the BBHI participants, suggested that in 10 years almost one third of our cohort will be newly diagnosed with a neurological or psychiatric disease (see **Figure 3**; Von Campenhausen et al., 2005; Bermejo-Pareja et al., 2008; Garre-Olmo et al., 2010; Kirkbride et al., 2011; Pradas et al., 2013; Petersen et al., 2014; Vila-Corcoles et al., 2014). Consequently, affording us the possibility to study lifestyles and biological determinants of brain health with sufficient signal.

#### Surveys

The initial questionnaire consists of questions pertaining to demographic, socio-economic and anthropometric information, as well as the presence of medical diagnosis and other health risk factors (e.g., smoking status, alcohol consumption).

To evaluate self-perceived general health, pain, cognitive and mental complaints we used the Patient-Reported Outcomes Measurement Information System (PROMIS) of global health and pain interference short forms, the Neuro-Qol cognitive function, and the Patients health questionnaire for depression and anxiety (Ader, 2007; Kroenke et al., 2009; Cella et al., 2012). Living habits in nutrition, physical activity and sleep were explored using items from the Mediterranean Diet Adherence Scale (Schröder et al., 2010), a physical activity scale, and the Jenkins Sleep Evaluation Questionnaire (JSEQ, Jenkins et al., 1988) respectively. Finally, items of the Ryff scale (Ryff, 1995), which assesses psychological well-being, are used to measure the dimensions of “purpose in life” and “personal growth.”

In order to assess the test–retest reliability of this first questionnaire we readministered it with a mean interval of 58 ± 3 days. 1,835 participants returned the re-administered questionnaire. The Interclass Correlation Coefficient (ICC) revealed a very good test-retest reliability for every sub-scale (all *r* ≥ 0.80; all *p* < 0.01) as well as for the whole questionnaire (*r* = 0.89; *p* < 0.01) indicating that it represents a valid and relatively time-inexpensive instrument for multi domain screening.

After this first questionnaire participants are asked to fill out further validated questionnaires that probe in more detail specific metrics associated with each of the 7 hypothesized pillars of brain health. Launched between July and December 2017, these include the Mediterranean Diet Adherence screener (MeDAS, Schröder et al., 2010), the Godin–Shepard Leisure time Physical Activity questionnaire (Godin and Shephard, 1997), the Depression, Anxiety and Stress Scale (DASS; Brown et al., 1997), a Cognitive Reserve Questionnaire (Rami et al., 2011), the Engaged Living Scale (ELS; Trompetter et al., 2013), the Sense of Coherence scale (SOC; Antonovsky, 1993), the Ryff scale of Psychological well-being (Ryff, 1995), the Self Health Horizon Questionnaire (SHH-Q; Düzel et al., 2015), the Pittsburg Sleep Quality index (PSQI; Buysse et al., 1989) and the Lubben Social Network scale (LSNS; Lubben, 1998).

**FIGURE 4:**
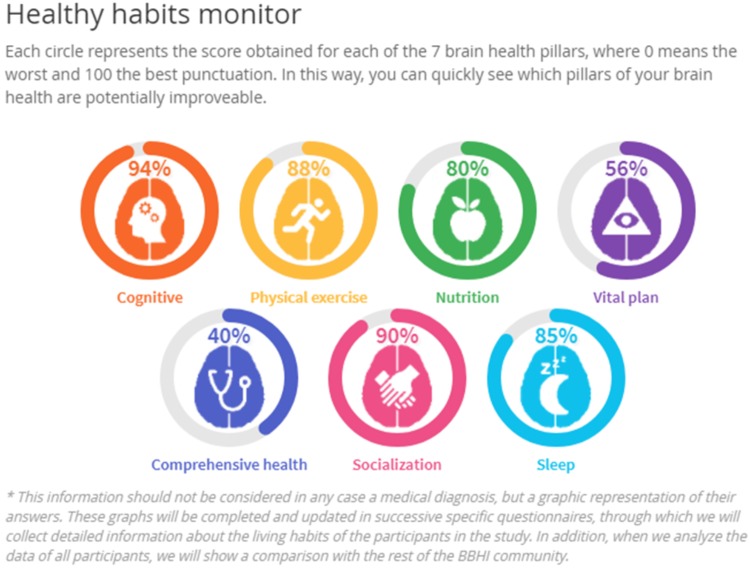
An illustration of the graphical feedback (‘Healthy Habits Monitor’) of an individual participant’s responses to questionnaires regarding the 7 hypothesized pillars of brain health.

Upon completion of the first questionnaire, a graphical representation of the responses is provided to each participant. The goal is to offer the participant a user friendly practical feedback as a first step toward a ‘personal brain health index.’ The graphic is termed the ‘Healthy Habits Monitor’ (**Figure 4**) and is regularly updated with the completion of additional questionnaires that address specific aspects of the brain health. Thus participants can see the evolution of their ‘healthy habits’ over the course of the study.

### Phase II: In Person Assessment

#### Sub-cohort Selection Criteria

**FIGURE 5:**
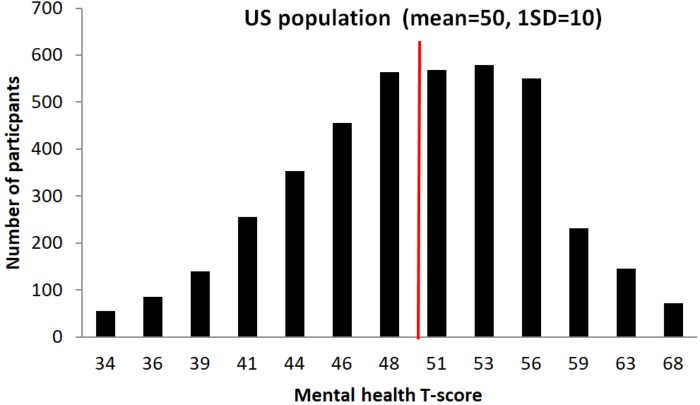
Graphical representation of participant’s *T*-scores distribution in the Mental Health sub-questionnaire, and comparison with US population. An independent samples *t*-test showed that the scores in the Mental Health index of all BBHI participants (*N* = 4206; mean = 49.8; *SD* = 6.9) do not differ significantly from reference scores of the general US population (values taken from PROMIS Global health scoring Manual: mean = 50; *SD* = 10; *t* = 1.7, *p* = 0.10).

For phase II, 1,000 participants from the BBHI main cohort, based on the information gathered in phase I have been invited to participate. Selection criteria for phase II is based on the “Mental Health” subscale (Hays et al., 2009) from the global health items of the PROMIS (Ader, 2007). This sub-scale evaluates mental-health by asking for self-perceived quality of life, social activities and relationship, emotional and cognitive problems. **Figure 5** illustrates the distribution of responses from the BBHI cohort in this scale compared to the US general population.

**FIGURE 6:**
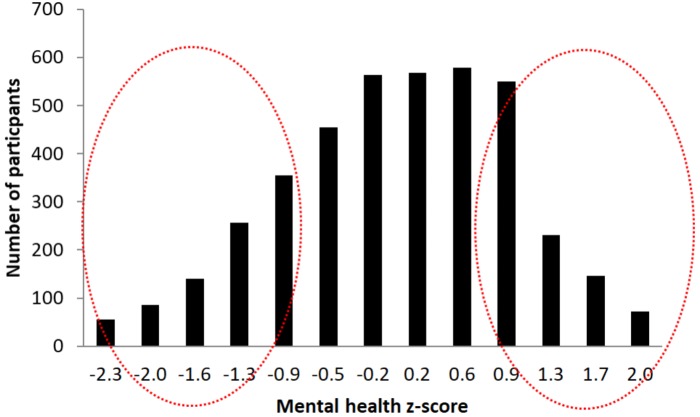
Graphical representation of participant’s *z*-scores distribution in the Mental health sub-questionnaire. The red circles marks the two selection groups at the two extremes of the distribution.

In order to select participants for the second phase of the study we calculated their scores in this sub-scale, transformed in standardized *z*-score, analyzed the resulting distribution (**Figure 6**), and selected, stratifying for age and sex, 500 participants from each of the two extremes of the distribution (1,000 in total). These two extremes represent participants with best and worst perceived mental-health of the cohort. The rationale for these selection criteria is to afford us the greatest possible signal to detect differences in biological correlates of brain health by comparing those with the best and worst self-reported brain/mental health. Indeed lower scores in these measures have been associated in previous studies with a variety of conditions like pain behavior, pain interference, fatigue, anxiety, depression, physical functioning, satisfaction with participation in social roles, and satisfaction with participation in discretionary social activities (Rothrock et al., 2010).

#### Assessments

##### Medical and physical exam

Medical and physical examination procedures were approved by the institution review board of the Institut Guttmann. Upon singing informed consent the participants undergo a medical evaluation and screening. This exam gathers medical personal and family history, social history, educational history, review of systems, and includes a medical exam, a cardiopulmonary exercise test (CPET), an evaluation of gait and balance, and a dual task performance test (Manor et al., 2016, 2018). Biological samples, initially focusing on blood and stool, are collected for laboratory tests and the determinations of biomarkers. Further blood samples are stored for genetic and epigenetic determinations. Biochemical, proteomics and metabolomic profiling will be carried out in plasma samples.

##### Cognitive assessment

The neuropsychological assessment comprises both paper and pencil, and computerized tests, administered in two separate sessions.

Paper and pencil assessment consists of a battery of well-established neuropsychological tests, exploring the different cognitive functions, while the computerized cognitive assessment includes four task used in the Cognition, Brain, and Aging study (COBRA; Nevalainen et al., 2015) evaluating processing speed and working memory.

The paper and pencil neuropsychological assessments evaluate general/fluid intelligence (WAIS-IV Matrices, Weschler, 2008), premorbid intelligence (National Adult Reading Test; Nelson and Willison, 1991) vocabulary (semantic verbal fluency, Peña-Casanova, 2005), selective attention (Cancelation test WAIS-IV; Weschler, 2008), executive functions (Trial making test B, phonemic verbal fluency; Reitan and Wolfson, 1985; Peña-Casanova, 2005) and working memory (Digit backward, Letter number sequencing), episodic memory (RAVLT; Rey, 1958), processing speed (digit symbol substitution; Weschler, 2008), motor and visuo-motor speed (finger-tapping, TMT-A; Reitan and Wolfson, 1985; Shimoyama et al., 1990), visuo-spatial abilities (Block design; Weschler, 2008).

##### Magnetic Resonance Imaging

Magnetic Resonance Imaging (MRI) data acquisition is undertaken using a 3 Tesla Siemens PRISMA scanner and a 32-channel head coil.

The MRI session lasts approximately 1 h and includes accelerated multiband sequences adapted from the Human Connectome Project (D) and provided by the Center of Magnetic Resonance Research (CMRR) at the University of Minnesota. The protocol includes several high resolution structural T1-, T2-weighted and a FLAIR scans, which will allow us to quantify longitudinal changes in cortical and subcortical volume, myelination, white matter integrity and vascular disease.

High-resolution multishell diffusion-weighted MRI (1.5 mm × 1.5 mm × 1.5 mm, 100 directions) is obtained to further characterize tissue integrity in white matter, where the anisotropy of water diffusion is known to capture the geometry and state of myelin sheaths along axonal tracts, but also within the cortex.

High resolution resting state functional MRI (fMRI) (2 mm × 2 mm × 2 mm and TR = 0.8 s) is acquired to study lifespan longitudinal changes in both whole-brain functional networks and patterns of local cortico-cortical coupling.

A second fMRI scan is acquired during a working memory and memory encoding tasks. To this end, two subgroups are randomly selected out of our Phase-II sample, each assigned to one memory task to avoid overloading the MRI protocol.

Finally, an advanced high resolution pseudo-Continuous Arterial Spin Labeling (pCASL) sequence is acquired to identify small focal lesions and quantify cerebral blood flow (CBF) and arterial transit time (ATT) in cortical and subcortical structures.

The brain anatomical MRI’s are reviewed with a neuroradiologist in order to generate a first report that might identify actionable findings and offer some return of results to participants.

##### Electroencephalography (EEG)

Thirty two channel EEG is acquired using the Enobio 32-channel EEG cap from Neuroelectrics (Barcelona, Spain) during rest (eyes open and eyes closed) and during the performance of an auditory Oddball task and a Flanker task. These tasks are used to collect event-related potentials (ERPs) to assess the neurophysiological response to conflict resolution monitoring and signal detection. Some changes in ERPs’ components elicited by these tasks, e.g., the P300 in the Oddball tasks, have been found to relate with information processing and age-related cognitive decline (see van Dinteren et al., 2014 for a meta-analysis). In addition, further electrophysiological EEG substrates are collected and analyzed such as frequency analysis oscillatory power bands and coherence, an index of the synchrony between cortical regions.

Neuroelectrics Instrument Controller software is used for initial EEG analysis in order to generate a first report that might identify actionable findings and offer some return of results to participants.

##### Transcranical Magnetic Stimulation and EEG

Transcranial magnetic stimulation combined with EEG allows the characterization of cortical excitability and reactivity (Pascual-Leone et al., 2011; Shafi et al., 2014; Farzan et al., 2016). Single pulse and paired pulse TMS using a Medtronic MagPro X100 stimulator, combined with high density TMS compatible EEG system (BrainProducts Brain ActiChamp, Gilching, Germany), are applied over 5 stimulation sites: primary motor cortex (M1), left and right inferior parietal lobule, and left and right lateral dorsolateral prefrontal cortex. These target areas are defined based each individual’s brain MRI and targeted using frameless stereotaxy. The targeting of these latter regions is based on the fact that they comprise nodes of two brain networks highly sensitive to the effects of aging, namely the fronto-parietal and the default mode network (DMN, Andrews-Hanna et al., 2007; Sala-Llonch et al., 2015; Damoiseaux, 2017), which is also critically affected in early stages of AD (Sheline and Raichle, 2013). Motor evoked potentials (measured via electromyography at the hand muscle) from M1 stimulation and TMS-evoked potentials (TEPs) are recorded from non-motor regions.

##### Participants Feedback and Incidental Findings Report

Once finished the in-person assessment an initial review of results is done by expert personal and supervised by a physician in order to identify actionable and incidental findings. Additionally, a brief report is developed that is subsequently sent to the participant with an explanation of which tests were performed, the result, and a brief overview of the significance of each test. Participants are informed about the lack of clinical value of this report and that this assessment does not substitute any medical or clinical assessment planned or previously done. Incidental medical findings are evaluated by the designated physician and communicated to participants as soon as an abnormality is detected during the assessments. Expert staff will be consulted as needed to assist with the interpretation of the results and the recommendations for follow-up (e.g., radiologist, neurologist/neurosurgery specialist, cardiologist, sleep physician,…). If it is determined that the participant would benefit from medical follow-up, he or she will be instructed to contact their general practitioner who will be provided with a report of the relevant findings. If there is concern for the participant’s immediate health, they will be referred to emergency medical care as needed.

### Phase III: Randomized Control Trial of a Multi-Dimensional Lifestyle Intervention

The third phase of the study is aimed to start in 2019 and will consist of a 6-month multi-dimensional intervention, administered via an Information and Communication Technology (ICT)-based platform. Five hundred participants from the general cohort who did not participate in phase II will be invited to participate. Phase III will be a randomized-control trial (RCT) of a multidomain intervention of remotely supervised coach-led lifestyle habits. A control group will consist of participants who receive education regarding healthy lifestyles but no coaching. All participants will undergo the in-person evaluations described in phase II at baseline and following the 6-month intervention. All participants will be monitored using mobile technologies to assess adherence to the multi-dimensional intervention and capture relevant metrics of physical activity, diet, sleep, etc.

#### Multi-Dimensional Intervention: Rationale and Characteristics

The multi-dimensional intervention will include cognitive training, physical exercise, adherence to the Mediterranean diet, maintenance of social activities, healthy sleep habits, and participation in mindfulness sessions. Several recent reviews and meta-analyses have demonstrated the weaknesses and inconsistencies of single physical, nutritional, or cognitive interventions (Brasure et al., 2018; Butler et al., 2018a,b) for the promotion of cognitive fitness and brain health. Indeed, the multi-factorial etiology of neurodegenerative diseases may necessitate the use of multi-domain interventions (Mangialasche et al., 2012). Accordingly, the need to evaluate the effects of multi-dimensional interventions is present (Kivipelto et al., 2013; Vellas et al., 2014; Kulmala et al., 2018) and a number of recent studies have assessed their effects. The Finnish Geriatric Intervention Study to Prevent Cognitive Impairment and Disability (FINGER; Kivipelto et al., 2013; Kivipelto et al., 2018) explored the effects of a multi-dimensional intervention composed of cognitive training, physical exercise, diet and monitoring of cardiovascular risk factors in preventing cognitive decline in at risk people as measured by the Cardiovascular Risk Factors, Aging, and Incidence of Dementia (CAIDE) score. After a 2-year intervention results showed a small benefit of the intervention in global cognitive functioning (Ngandu et al., 2015; Rosenberg et al., 2018).

Following the same logic Vellas et al. (2014) assessed in the Multidomain Alzheimer Prevention Trial (MAPT) the efficacy of: (1) a single domain supplementation with omega-3 fatty acid; (2) a multi-dimensional intervention including nutritional counseling, physical exercise and cognitive stimulation; or (3) a combination of the two. Initial data showed no significant effects of any of the interventions on cognitive decline over 3 years (Andrieu et al., 2017). However, a secondary analysis of the data narrowed to high-risk people using similar criteria of the FINGER study (i.e., CAIDE ≥ 6) found benefit of the multi-domain intervention in some cognitive outcomes (Chhetri et al., 2018).

Two important lessons can be derived from these results. Firstly, they highlight the importance of early interventions to prevent cognitive decline. That is, as previously mentioned (see Introduction), changes in the brain begin decades prior to the onset of symptoms and consequently, lifestyle interventions may be necessary across the whole lifespan, long before these changes commence.

Second, a large number of sensitive baseline measures are fundamental to detect true effects of lifestyle interventions on cognitive decline and prevention of neurodegenerative diseases, and fully explain and understand the different age-related trajectories (Nilsson and Lövdén, 2018).

Phase III of the BBHI aims to fill these gaps in knowledge through a multi-domain lifestyle intervention, during mid-life with a large number of baseline and post-intervention metrics sensitive to differences in brain health. Prior to launching, this third phase of the BBHI will be registered in clinicaltrials.gov.

All the intervention will be ICT-based, designing and developing a solution consisting on two main parts: (1) a mobile application (the BBHI app) that participants will download; and (2) a web-based portal for coaches. The BBHI app will allow us to administer, coach and monitor the different domains of the intervention program. Users will have access to the different modules (addressing the defined 7 brain health pillars) and the web-based portal will allow coaches managing and tracking their performances in these different domains. This *ad hoc* ICT-based developed solution will allow us to have all monitored data stored in our Data Warehouse, structured and ready for analysis.

At the time of writing the exact details of the Phase III protocol are under discussion with the International Scientific Advisory Board Members of the study. Consequently, the exact protocols for each intervention will be guided by the most up to date literature on each topic at the time of the phase launch. Notwithstanding, there is consensus regarding the general lines of the intervention, which will include the following modalities;

*Cognitive training*, administered by the BBHI app, will be developed in-house based on previous successful intervention studies (Schmiedek et al., 2010; Schweizer et al., 2011; Wolinsky et al., 2011; Sandberg et al., 2014). It includes tasks that engage different cognitive domains as attention (e.g., visual search), speed of processing (e.g., perceptual speed task), set shifting (e.g., task switching), inhibition (e.g., Stroop task), working memory (e.g., dual n-back task), reasoning (e.g., sequencing task) and memory (visual span and recent probe task*).* As the majority of cognitive training studies we will use a multi-domain approach, that have often reported better effects over-time than single-domain cognitive training (Cheng et al., 2012; Lampit et al., 2014).

*Physical exercise*, monitored by the BBHI app and wrist-based heart rate technology. A recent systematic review has shed light on the optimal dosages for consistent exercise-mediated improvements in aging (Gomes-Osman et al., 2018). In line with this approach the exact frequency, intensity and type of our exercise intervention will be determined by the extant literature at the time of phase launch.

*Nutrition*, where participants will be asked to follow a typical Mediterranean diet (Lourida et al., 2013), and their adherence to this dietary prescription will be assessed periodically by a questionnaire (Schröder et al., 2010) administered via BBHI app.

*Healthy sleep habits*, which will be guided by recommendations from Global Council for Brain Health^4^ and monitored by the BBHI app.

Finally, participants will be asked to participate in group sessions directed to help them to focus on what is important for them and live a values oriented life, using technique that include meditation and mindfulness (Franquesa et al., 2017).

#### Coaching and Monitoring

Monitoring and control of participant adherence to a multi-dimensional intervention has been highlighted as a fundamental methodological issue faced in previous studies (Kivipelto et al., 2013; Vellas et al., 2014). Numerous recent studies evaluating health coaching programs stress the relevance of monitoring and coaching (Dejonghe et al., 2017) to increase participant motivation and adherence (Barbera et al., 2018). Nevertheless, there is still a research gap in regard to feasibility, scalability, and long-term effectiveness.

Although health coaching can be seen as an interpersonal process, where communication and relation between the coach and the user are central aspects, ICT-based solutions offer very interesting opportunities, in both measurement as well as intervention (Rutjes et al., 2017; Barbera et al., 2018). One of the main advantages of coaching programs supported by technology is the possibility of seamlessly measuring behavioral data, thanks to devices and wearables which provide objective data about performance. Thanks to the potential of large datasets to provide learning opportunities via the implementation of artificial intelligent processes, the prediction of the effectiveness of each intervention can be calculated.

In our proposal, each coach will be responsible for the individualization and follow-up of the multi-dimensional activity programs. This will be implemented remotely with the use of ICT technologies (Simkin-Silverman et al., 2011; Barbera et al., 2018) and based on the SMART Goals (Bovend’Eerdt et al., 2009), Reinforcement Theory of Motivation (Cameron and Pierce, 1994) and Social Comparison (Festinger, 1954) theories.

### Data Managing

BBHI is an ICT-based study, where technology plays a key role in the collection and management of the data. The data generated via online questionnaires is transferred using secure procedures and stored in a cloud server, which assures full-time access to data as well as the necessary security protocols. Encrypted communication based on TLS (Transport Layer Security) is used whenever personal information is transferred between systems. The collected data are anonymized and kept confidential to protect the privacy of the participating users, fulfilling all the requirements stated by the Spanish Organic Law and EU regulations.

## Anticipated Results

### Limits of Detection and Statistical Analysis

#### Phases I and II

Considering the sample size of participants in phase I we calculated the relative risk of being exposed to a “brain health related” factor (or combination of factors) by comparing the estimated incidence rate of any neurological and neuropsychiatric diseases predicted in the Catalan population and the incidence predicted in a certain % of the cohort exposed to a protective factor (see Dartigues et al., 1992; Cyceron, 2003).

Assuming α risk of 0.05, a β risk of 20%, and a 5% per year drop out, after 5 years we will be able to identify a protective factor that represents a relative risk of 0.77, if 30% of participants are exposed to it. If the number of participants exposed is 20% the minimum relative risk identifiable is 0.73 and for an amount of 10% it is 0.66.

For the in person assessment (phase II), where 1,000 volunteers will be examined in-person, we will be able to detect a 0.61 relative risk if 30% of participants are exposed, 0.55 relative risk for 20% exposed, and 0.44 relative risk for 10% exposed.

Based on evidence from previous studies that look at the association between the reduction in the incidence of various neurological and neuropsychiatric diseases and different lifestyles these results indicate that at 5 years we will have sufficient power to detect factors related with brain health maintenance in our sample (La Rue, 2010; Baglioni et al., 2011; Kuiper et al., 2015; Frankish and Horton, 2017). Note however, that the main goal of the phase II of BBHI is descriptive, aiming for a deep phenotyping characterization of the biological markers of brain health.

#### Effects of Intervention in Phase III

A recent meta-analysis (Zhu et al., 2016) proposed that the estimated combined effect size of physical and cognitive interventions, presented as Cohen’s *d*, is 0.29. In our study, considering α = 0.05, a β = 10 and an annual drop-out of 5%, we will be able to detect in a pre-post repeated measure ANOVA on the different outcomes (e.g., Flanker effect) between two groups of 250 participants a small effect *d* = 0.17.

#### Principal Statistical Analysis

Principal statistical analyses to be performed will include mainly linear regression models in within-subjects and between-groups experimental designs. We may require applying non-parametric statistics in certain cases.

We will also use exploratory data mining techniques like clustering and classifiers in order to identify groups of similar participants and explore the relation between different biologic and lifestyles variables and the different outcomes.

The final aim of this study is to investigate and identify those factors that can promote Brain Health. Considering the binary nature of the end-point of this study (phase I and II), the analysis within the study population will be based on the calculation of odds ratios, hazard ratios and relative risks of exposure to protective factors on the *a priori*-defined 7 pillars of interest. All analyses will be performed correcting for covariate variables (e.g., age, sex, education, income, etc.)

### Pitfalls, Artifacts and Troubleshooting

This study presents various pitfalls and limitations. Similar to all long-term longitudinal studies, participant drop-out is a crucial pitfall that needs to be addressed. In order to minimize participant drop-out we aim to keep participants engaged via strategies described previously (see “BBHI Launching and Recruiting” and “The BBHI Community”). Briefly, we maintain regular communication with participants via social media, periodic public conferences, dissemination of information on recent findings, organization of public events, as well as periodically inviting participants to face to face meetings with the BBHI research and leadership team.

Given the longitudinal nature of the study, another possible pitfall is the possibility for variability in data collection due to instructor bias. To minimize this risk detailed protocols and extensive training is performed by each new member of the research team. Additionally, periodic data quality checks are performed to ensure the quality and reproducibility of the data being collected.

Lastly, as discussed in Section “Characterization of the Cohort,” our cohort is currently not fully representative of the general population for gender (over-representation of women) and educational status (higher than general population). We aim to correct this through ongoing, targeted participant recruitment. Nevertheless, these biases will be considered when analyzing and interpreting and the data.

## Conclusion

The BBHI is a large longitudinal cohort study being conducted in Spain, focused on the promotion of brain health in middle aged adults. The study’s aims are to investigate the biological, environmental and social determinants of brain health maintenance, and whether a multi-dimensional intervention program, focused on life-style habit changes, can have short and long terms effects on cognitive functioning and reduces the incidence of neuropsychiatric brain diseases.

The BBHI will allow us to combine the findings from both a prospective cohort analysis and a randomized-controlled intervention, conducted with the same harmonized measures and outcomes. In line with recent biosocial models of network medicine (Greene and Loscalzo, 2017), the three phases of the BBHI with relate biologic evidence with environmental and social analyses, and enable a deeper understanding of factors promoting healthy aging. Beyond a holistic elucidation of health and disease in aging, the BBHI will investigate if an individualized multi-dimensional intervention program can enhance cognitive functions and reduce the incidence of new future diagnoses. Given the complex interactions of the determinants of individual pathophenotypes (Kivipelto et al., 2013), multi-dimensional interventions and their interactions may yield the best response.

The broad and favorable response to the BBHI, with fast recruitment and high retention rates of a very active cohort, reflects the interest and timeliness of the topic of brain health. Female (66%) and higher-educated (70%) persons are overly represented in the BBHI cohort. This, however, is in line with other longitudinal studies ongoing in Spain (Olazarán et al., 2015). The over-representation of these demographics, possibly reflects the advanced literacy required to complete the surveys (Olazarán et al., 2015) or the increased education on brain health among more educated individuals. Indeed, gender differences in the utilization of health care suggest that women tend to use diagnostic and preventative medicine more than men (Gómez Gómez, 2002).

## Ethics Statement

This study was carried out in accordance with the recommendations of the “Unió Catalana d’Hospitals” with written informed consent from all subjects. All subjects gave written informed consent in accordance with the Declaration of Helsinki. The protocol was approved by the ‘Unió Catalana d’Hospitals.’

## Author Contributions

AP-L, DB-F, and JT participated in the initial conception of the design of the project. GC, JS, DM, and TM made substantial contributions to the actual design and implantation protocol. GC and JS participated actively in the data collection and analysis. TM, DM, JT, DB-F, and AP-L contributed to the interpretation of the results. GC drafted the article and all other authors made critical revisions, introducing important intellectual content. Each author gave final approval of the submitted version.

## Conflict of Interest Statement

AP-L serves on the scientific advisory boards for Neuronix, Starlab Neuroscience, Neuroelectrics, Constant Therapy, and Neosync; and is listed as an inventor on several issued and pending patents on the real-time integration of transcranial magnetic stimulation with electroencephalography and magnetic resonance imaging. The remaining authors declare that the research was conducted in the absence of any commercial or financial relationships that could be construed as a potential conflict of interest.

## Footnotes

1 https://bbhi.cat

2 https://bbhi.cat

3 https://club.bbhi.cat

4 https://www.aarp.org
